# The Interaction of the Chemotherapeutic Drug Chlorambucil with Human Glutathione Transferase A1-1: Kinetic and Structural Analysis

**DOI:** 10.1371/journal.pone.0056337

**Published:** 2013-02-27

**Authors:** Michael Karpusas, Irine Axarli, Lykourgos Chiniadis, Athanasios Papakyriakou, Kostas Bethanis, Katholiki Scopelitou, Yannis D. Clonis, Nikolaos E. Labrou

**Affiliations:** 1 Physics Laboratory, Department of Science, Agricultural University of Athens, Athens, Greece; 2 Laboratory of Enzyme Technology, Department of Agricultural Biotechnology, Agricultural University of Athens, Athens, Greece; 3 Chemical Biology Laboratory, Institute of Physical Chemistry, NCSR “Demokritos”, Athens, Greece; Massachusetts Institute of Technology, United States of America

## Abstract

Glutathione transferases (GSTs) are enzymes that contribute to cellular detoxification by catalysing the nucleophilic attack of glutathione (GSH) on the electrophilic centre of a number of xenobiotic compounds, including several chemotherapeutic drugs. In the present work we investigated the interaction of the chemotherapeutic drug chlorambucil (CBL) with human GSTA1-1 (hGSTA1-1) using kinetic analysis, protein crystallography and molecular dynamics. In the presence of GSH, CBL behaves as an efficient substrate for hGSTA1-1. The rate-limiting step of the catalytic reaction between CBL and GSH is viscosity-dependent and kinetic data suggest that product release is rate-limiting. The crystal structure of the hGSTA1-1/CBL-GSH complex was solved at 2.1 Å resolution by molecular replacement. CBL is bound at the H-site attached to the thiol group of GSH, is partially ordered and exposed to the solvent, making specific interactions with the enzyme. Molecular dynamics simulations based on the crystal structure indicated high mobility of the CBL moiety and stabilization of the C-terminal helix due to the presence of the adduct. In the absence of GSH, CBL is shown to be an alkylating irreversible inhibitor for hGSTA1-1. Inactivation of the enzyme by CBL followed a biphasic pseudo-first-order saturation kinetics with approximately 1 mol of CBL per mol of dimeric enzyme being incorporated. Structural analysis suggested that the modifying residue is Cys112 which is located at the entrance of the H-site. The results are indicative of a structural communication between the subunits on the basis of mutually exclusive modification of Cys112, indicating that the two enzyme active sites are presumably coordinated.

## Introduction

Glutathione transferases (GSTs) are a family of Phase II detoxification enzymes that catalyse the conjugation of the tripeptide glutathione (γ-Glu-Cys-Gly, GSH) to a wide variety of electrophilic compounds. Human cytosolic GSTs, on the basis of their amino acid sequence, can be divided into the following eight classes: *alpha*, *mu*, *pi*, *sigma*, *theta*, *zeta*, *kappa* and *omega* [1]–[5]. Structural studies have shown that GSTs are dimeric enzymes with each subunit containing a GSH-binding site (G-site) and a second adjacent hydrophobic binding site for the electrophilic substrate (H-site) [2]. Amino acid variations of the H-site, among the different GST classes, determine substrate specificity.

GSTs are considered as a drug targets since specific isozymes are overexpressed in a variety of tumour cells [6]–[9]. The development of chemotherapy resistant tumour cells is a significant problem encountered in cancer chemotherapy. GSTs have been implicated in the development of resistance toward chemotherapy agents [7]–[9]. A possible origin for this problem appears to be an increase in the expression of total GST activity. It is possible that GSTs confer drug resistance by two distinct means: by direct inactivation (detoxification) of chemotherapeutic drugs and by acting as inhibitors of the mitogen-activated protein (MAP) kinase pathway [8]. Furthermore, GSTs have been identified as inhibitors of stress-activated kinase activities, thereby protecting cells against apoptosis in response to cellular stress from reactive oxygen species. In addition, it has been shown that GSTA1 suppresses activation of JNK (c-Jun N-terminal kinase) signalling by a pro-inflammatory cytokine and oxidative stress, thus indicating a possible protective role for GSTA1-1 against JNK-associated apoptosis [10].

The catalytic function of the GSTs is far from completely characterized [6]–[9]. Various electrophilic xenobiotics are used as substrates by GSTs. Electrophilic centres for GSH conjugation are found in areneoxides, aliphatic and arylic halides, in carbonyls, organonitro-esters, organic thiocyanates, including certain chemotherapeutic drugs [3], [5], [6]. Identification of the GST-mediated pathway for drug cleavage has been useful for elucidating the mechanism of metabolic biotransformation of compounds that have been brought forward for clinical studies and, furthermore, enables the design of new molecules that exhibit improved efficacy and pharmacokinetic characteristics [11], [12]. Chlorambucil (CBL) is a nitrogen mustard alkylating drug that is mainly used in the treatment of chronic lymphocytic leukemia [13]. Human GSTA1-1 (hGSTA1-1) is an effective catalyst for chlorambucil conjugation with GSH [14]–[16]. It has been reported that combined expression of GSTA1-1 and multidrug resistant protein 1 (MRP1) or multidrug resistant protein 2 (MRP2) in MCF-7 cells confers resistance to CBL [14], [17]. Expression of GSTA1-1 alone in MRP-deficient MCF-7 cells failed to confer resistance to CBL, since the CBL-GSH conjugate rapidly accumulates to levels that completely inhibit GSTA1-1 catalysis of CBL conjugation.

The present work combines kinetic, crystallographic and computational dynamics approaches for studing the interaction of the chemotherapeutic drug CBL with hGSTA1-1. The results of the present study provide an insight at the molecular level on the mechanism of cancer cell resistance against CBL therapy.

## Materials and Methods

### Materials

GSH, 1-chloro-2,4-dinitrobenzene (CDNB), CBL and all other reagents and analytical grade chemicals were obtained from Sigma-Aldrich Co (USA).

### Methods

#### Cloning, expression and purification of hGSTA1-1

Cloning, expression and purification of hGSTA1-1 was carried out as described by Axarli et al. (2009) [9]. Before kinetics analysis the purified enzyme was incubated with 10 mM dithiothreitol to ensure complete reduction of cysteine residues, followed by extensive ultrafiltration against potassium phosphate buffer (20 mM, pH 7.0). Before crystallization the enzyme was subjected to dialysis against Tris/HCl buffer (10 mM, pH 7.0).

#### Assay of enzyme activity and protein

GST assays were performed by monitoring the formation of the conjugate between CDNB and GSH at 340 nm (ε = 9.6 mM^−1^·cm^−1^) according to a published method [18]. Alternatively, GST assays using CBL and GSH were performed 37°C by measuring the rate of chloride ion released using the methods of Skopelitou and Labrou (2010) [19]. Observed reaction velocities were corrected for spontaneous reaction rates when necessary. Enzyme-dependent catalysis was determined by subtracting observed rates for conjugate formation in the absence of GSTA1-1 from observed rates in the presence of GSTA1-1. Kinetic constants were calculated from the initial velocities of enzyme-dependent conjugate formation fitted to the Michaelis-Menten equation using the computer program GraFit [20]. All initial velocities were determined in triplicate in buffers equilibrated at constant temperature. One unit of enzyme is defined as the amount of enzyme that produces 1.0 µmole of product per minute.

#### Viscosity dependence of kinetic parameters

The effect of viscosity on k_cat_ was studied at 37°C, in 0.1 M potassium phosphate buffer, pH 6.5, containing variable glycerol concentrations. Viscosity values (η) were calculated as described in [21], [22]. Glycerol does not induce changes in the enzyme secondary structure as detected by far-UV difference spectroscopy. Furthermore, glycerol does not have any inhibitory effect on catalysis.

#### X-ray crystallography

For the crystallization, protein (19.7 mg/mL) was incubated with CBL at a 1∶3 molar ratio and the solution was mixed with equal volume of reservoir solution containing 17–20% PEG 2000, 0.1 M Tris-HCl pH 7.5, 0.1 mM β-mercaptoethanol. Hanging or sitting drops of the mixture were placed over the reservoir solution and crystals were grown by vapor diffusion at 20°C. The crystals appeared after 2–3 days, but were used for data collection after 2–3 months. For cryoprotection, crystals were rapidly transferred to a solution of 30% PEG 2000, 0.1 M Tris-HCl pH 7.5 and were flash-frozen in liquid nitrogen. X-ray diffraction data were collected at 110° K, using an RAXIS-IV detector mounted on a Rigaku rotating anode generator producing CuKα radiation (Rigaku-MSC, Woodlands TX). The data were processed with the HKL program package [23]. The crystal structure was solved by molecular replacement and difference Fourier map techniques. Crystallographic refinement was done with the CNS program package [24] and molecular graphics manipulations were done with the program Coot [25]. Additional crystallographic statistics are shown in Table 1. Atomic coordinates have being deposited in the RCSB Protein Data Bank with accession code 4HJ2.

**Table 1:** Crystallographic data collection and refinement statistics.

Space group	C2
a (Å)	99.00
b (Å)	93.53
c (Å)	51.42
β (°)	93.91
Resolution range (Å)	20.0–2.1
Unique reflections	23814
Completeness (%)^a^	87.2 (84.9)
I/σ(I)	13.16 (2.76)
R_merge_ b	5.0 (22.9)
R_fact_ (%)	21.5
R_free_ (%)^c^	25.8
Water molecules	299
Average B-value (Å^2^)	28.1
RMSD (bonds) (Å)	0.007
RMSD (angles) (Å)	1.346

(a) The values in parenthesis refer to the highest resolution shell (2.18–2.1 Å).

(b) R_merge_ = Σ_h_Σ_i_ |I_hi_-I_h_|/Σ_hi_I_hi_.

(c) R_free_ was calculated against 5% of the reflections removed at random.

#### Molecular Dynamics

The crystal structure of hGSTA1-1/GSH-CBL complex was used for the preparation of all simulation systems. GST residues Lys4–Phe220 were only considered for both monomers, whereas solvent molecules were removed. The missing residues Cys112–Glu115 (monomer B) were modeled by using the corresponding coordinates of monomer A, when superimposed with monomer B. Hydrogen and other heavy atoms were added using the XLEaP module of AMBER 9 [26]. Histidine protonation states were manually set by examining the surrounding microenvironment of each imidazole ring. Specifically, His8 and His143 were protonated at N^ε2^ due to their hydrogen bonding interaction with O^ε2^ of Glu59 and C = O of Leu148, respectively, while His159 was set to be protonated at N^δ1^ because of the observed N^ε2…^H–Ν^ε^ (Arg155) interaction. All other basic residues were protonated and all acidic residues were deprotonated. The modified PARM99SB force field parameters were applied to the protein atoms using XLEaP [27]. Ligand force field parameters were retrieved from the GAFF set and AM1-BCC atomic charges were calculated using the ANTECHAMBER module of AMBER [28].

Three simulation systems were employed for molecular dynamics: a) the apo hGSTA1-1 dimer lacking all ligand coordinates, b) the holo hGSTA1-1/GSH–CBL dimer and c) the hGSTA1-1/GSH model prepared by removing the coordinates of CBL atoms. In order to relax them from crystal-packing contacts and optimize the position of hydrogen atoms, a set of minimization rounds were carried out with the SANDER module of AMBER. First, 1000 minimization steps were performed in order to allow relaxation of hydrogen atoms only, then 1000 steps for the ligands and the residues with missing atoms, and finally, 3000 steps of conjugate gradient minimization restraining only the protein Cα atoms. Positional harmonic restraints were imposed accordingly using a force constant of 50 Kcal·mol^−1^·Å^−2^. The generalized Born solvation model GB^HCT^ was employed using a 16-Å cutoff for truncation of nonbonded interactions. Subsequently, the simulation dimmers were immersed in isometric truncated octahedra of pre-equilibrated TIP3P waters using a buffer of 8 Å and the appropriate number of chloride ions was added to neutralize the total charge. The PMEMD module of AMBER 9 was used for the molecular dynamics calculations. Numerical integration was performed with a 2-fs time step, and all bonds involving hydrogen atoms were constrained with SHAKE. Periodic boundary conditions were imposed by means of the particle-mesh Ewald method with an 8-Å limit for the direct space sum. Temperature and pressure controls were imposed using a Berendsen-type algorithm with coupling constants of 1 ps for the thermostat and 2 ps for the barostat. Prior to the production run, all systems were equilibrated as described below.

Initially, water molecules and ions were subjected to energy minimization for 1000 steps, of which 100 steps employed the steepest descent algorithm and 900 employed conjugate gradients. Protein atoms were restrained harmonically with a 50 Kcal·mol^−1^·Å^−2^ force constant. While all solute atoms were still restrained, the temperature was increased to 300 K through 20 ps of constant volume dynamics (NVT ensemble). A second round of 1000-step energy minimization using conjugate gradients was then carried out by restraining only the protein Cα atoms. Subsequently, all restraints were removed and the system temperature was increased to 300 K within six 10-ps rounds in the NVT ensemble. The systems were then equilibrated at 1 atm throughout 150 ps of constant pressure dynamics (NPT ensemble). Production runs were carried out in the NPT ensemble for a total of 10 ns each. The translational center-of-mass motion was removed every 2 ps, while trajectories were updated every single ps. Post-simulation analysis was performed using the PTRAJ module of AMBER and the trajectories were inspected in VMD 1.8.6 [28], [29].

#### Enzyme inactivation studies by CBL

Inactivation of hGSTA1-1 was performed in 1 mL of incubation mixture containing potassium phosphate buffer pH 7 (100 µmol), CBL (0.5–3.0 mM) and enzyme, (0.1 units). The rate of inactivation was followed by periodically removing samples (5–20 µL) for assay of enzymatic activity [30], [31]. Rate constants for the reaction exhibiting biphasic kinetics were calculated using the equation [32], [33]:
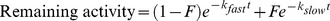
where F represents the fractional residual activity of the partial active enzyme intermediate. k_fast_ and k_slow_ are the rate constants for the slow and fast phase of the reaction. Analysis was achieved using the GraFit [20] computer program. K_D_ determinations were performed according to King et al. (1983) [32] and Wang et al., (1998) [33].

Inactivation studies of hGSTA1-1 by CBL in the presence of *S*-nitrobenzyl-GSH were performed in 1 mL of incubation mixture containing potassium phosphate buffer pH 7 (100 µmol), CBL, (2 mM), *S*-nitrobenzyl-GSH (1 mM) and enzyme (0.1 units).

#### MALDI-TOF MS

Enzyme samples (hGSTA1-1 and hGSTA1-1-CBL conjugate) were desalted with ZipTip μ-C18 (Millipore, USA). A saturated solution of sinapinic acid in water/acetonitrile (50/50, v/v, 0.1% trifluoroacetic acid) was used as the matrix solution. One µL of the sample and matrix mixture was spotted into a well of the sample plate and dried on the sample holder. Mass spectra were taken with a Voyager System 6322 (Applied Biosystems). Mass calibration was performed using bovine serum albumin, apomyoglobin and cytochrome c.

#### Modification of hGSTA1-1 with DTNB and maleimide

Determination of enzyme cysteine-SH groups with 5,5-dithio-bis-(2-nitrobenzoic acid) (DTNB) was based on the Ellman's method [35]. The assay was carried out in 100 mM potassium phosphate buffer, pH 7.6, in the presence of 1 mM DTNB. The resultant nitrophenyl anions from the reaction were quantified from the change of absorbance at 412 nm. Control incubation in the absence of hGSTA1-1 was taken to correct the above determinations for alkaline hydrolysis of DTNB. Modification of hGSTA1-1 by maleimide (1 mM) was carried out according to Lyon and Atkins (2002) [36].

#### Determination of the stability of CBL

The rate of decomposition of CBL in a buffer identical to that used in the inactivation studies (100 mM potassium phosphate buffer pH 7) was determined by following the time dependent release of chloride ions using the method of Skopelitou and Labrou, 2010 [19].

## Results

### The interaction of CBL with hGSTA1-1 in the presence of GSH

#### Kinetic analysis and the rate-limiting step of the CBL/GSH catalytic reaction

**Figure 1:**
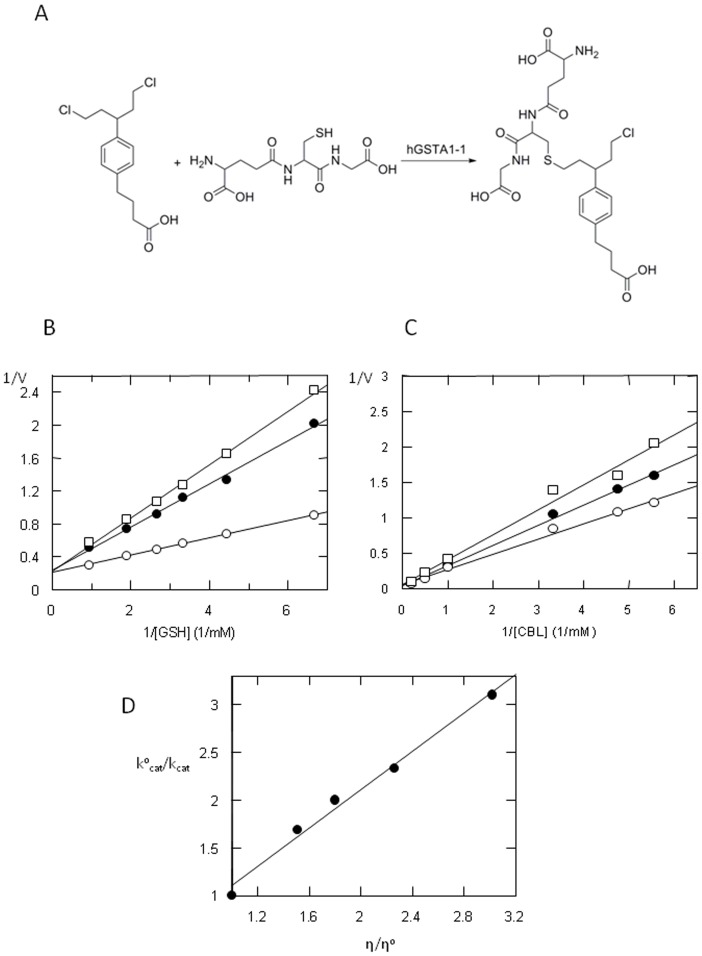
Kinetic analysis of the CBL/GSH reaction catalyzed by hGSTA1-1. (A): Reaction between CBL and GSH catalyzed by hGSTA1-1. (B): Initial velocity analysis with GSH as the variable substrate (0.15–1.05 mM) for several fixed concentrations of CBL (mM): 0.1, (□); 0.2, (•); 0.4, (○). (C): Initial velocity analysis with CBL as a variable substrate (0.02–0.5 mM) for several fixed concentrations of GSH (mM): 0.5, (□); 1.0, (•); 2.0, (○). (D): The effect of viscosity on turnover number. Plot of the reciprocal of the relative turnover number (k^o^_cat_/k_cat_) as a function of relative viscosity (η/η^o^) with glycerol as a cosolvent. Experiments were performed in triplicate and lines were drawn by least-squares regression analysis.

hGSTA1-1 is an effective catalysts for CBL conjugation with GSH (Figure 1A) [14], [37], [38] leading to the formation of the monoglutathionyl derivative of CBL but not the diglutathionyl, one [15], [16]. Whether GSH was used as a variable substrate with several fixed concentrations of CBL, an intersecting pattern of Lineweaver-Burk plot was obtained (Figure 1B), and with CBL as the variable substrate at fixed concentrations of GSH, an intersecting pattern was again obtained (Figure 1C), suggesting a sequential kinetic mechanism for this enzyme.

The viscosity effect on k_cat_ was investigated to determine the nature (physical or chemical event) of the rate-limiting step of the catalytic reaction (Figure 1D) [18], [39], [40]. The decrease of k_cat_ by increasing the medium viscosity shows that the rate-limiting step of the catalytic reaction is related to the product release or to diffusion-controlled structural transitions of the protein [41], [42]. In particular, a plot of the inverse relative rate constant k_cat_^o^/k_cat_, (k_cat_^o^ is determined at viscosity η^o^) versus the relative viscosity η/η^o^ should be linear with a slope equal to unity if a physical step is the rate-determining one, whereas a slope of zero indicates that a chemical reaction step is the rate limiting one [40]–[42]. The enzyme displayed a linear dependence with a slope of approx. 1.0 (slope = 1.01±0.06) (Figure 1D), suggesting that a physical step of the reaction that effects product release and/or structural transition is the rate-limiting determinant.

#### Crystallographic analysis

**Figure 2:**
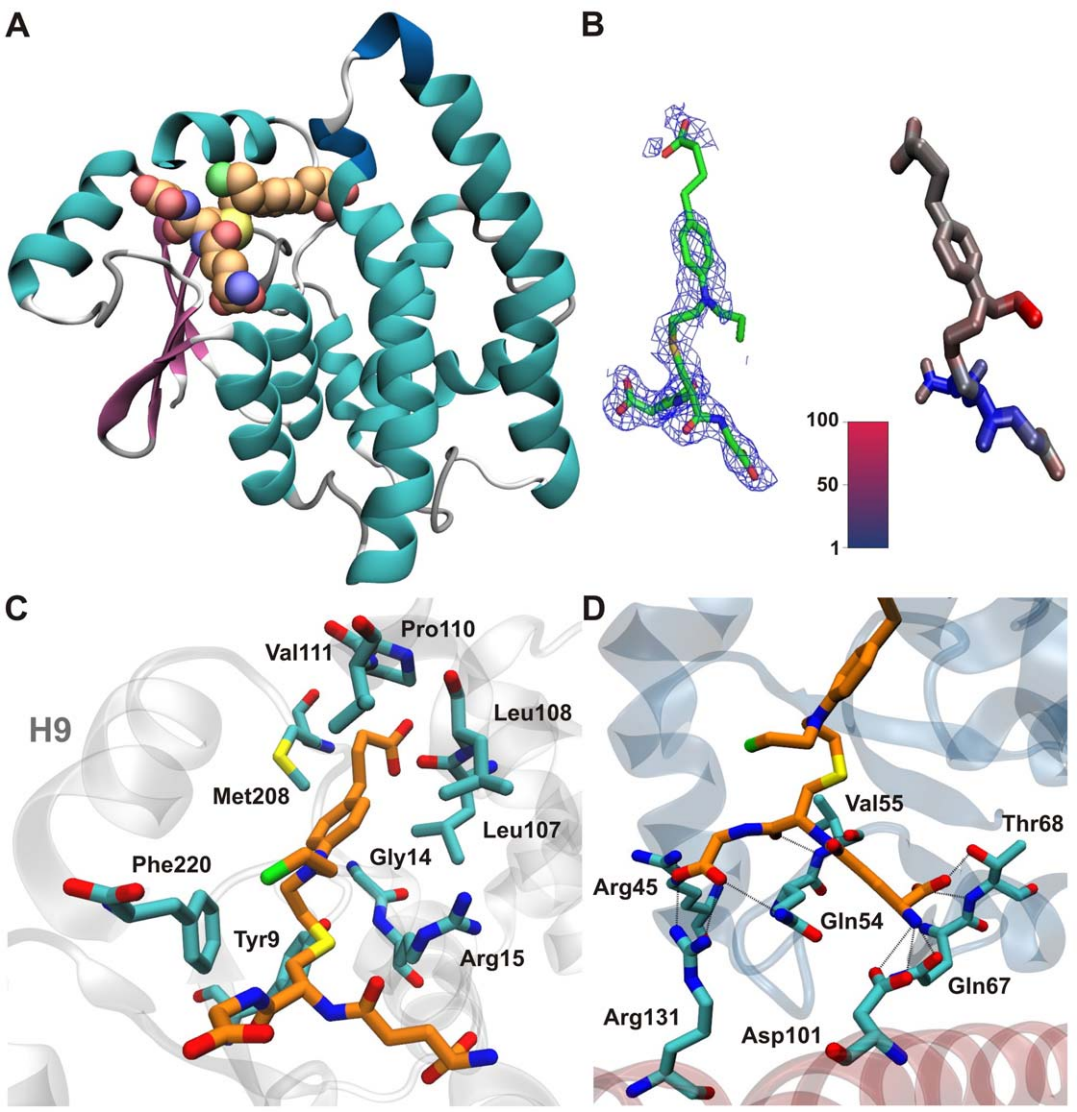
Crystal structure of hGSTA1-1/GSH–CBL complex. (A) Ribbon representation of monomer A with the GSH–CBL adduct atoms shown as van der Waals spheres and coloured orange for C, blue for N, red for O, yellow for S and green for Cl. (B) Representative 2Fo-Fc electron density map contoured at 1σ in the vicinity of bound GSH–CBL superimposed on refined crystal structure (left) in comparison with the simulated GSH–CBL adduct coloured according to the B-factor calculated from 10-ns molecular dynamics (right). (C) Residue-specific interactions of CBL moiety (orange licorice) in monomer A (cyan licorice). (D) Residue-specific interactions of the GSH moiety (orange licorice) from monomer A (blue ribbons) and monomer B (red ribbons).

**Figure 3:**
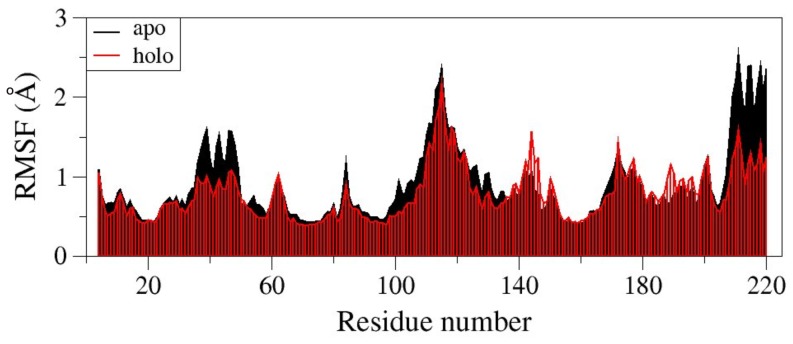
Root mean square fluctuations (RMSF) of hGSTA1-1 Cα atoms calculated for the apo (black) and the holo complex with GSH–CBL (red) from 10-ns molecular dynamics simulations. The graph shows the mean RMSF value of the two monomers.

The hGSTA1-1 enzyme was crystallized in the presence of CBL and the X-ray crystal structure was determined by molecular replacement at 2.1 Å resolution. The crystal form of hGSTA1-1 obtained is identical to a previously reported crystal form [43], [44]. Table 1 summarizes relevant crystallographic statistics. The final model includes hGSTA1-1 residue ranges 4–220 for monomer A (Figure 2A) and 3–221 for monomer B that fit satisfactorily into the electron density map. In addition, residues 112–115 of monomer B are not included in the model due to non-interpretable electron density. All residues are within the allowed regions of the Ramachadran diagram, with the exception of Gln47 which has well defined electron density and has been observed to adopt the same unusual conformation in other GSTA1-1 crystal structures [43]–[45].

Difference Fourier electron density maps indicate the presence of a GSH moiety in the G-site of the enzyme (Figure 2B). Even although GSH was not included in the crystallization solution, it was apparently still present in the protein sample used. It most likely originated from previous use in the elution step from the affinity chromatography step on GSH-Sepharose adsorbent. The GSH moiety is well ordered and adopts a conformation essentially identical to that observed in the previously reported GSTA1-1 structures [43]–[45]. Specifically it forms a salt bridge with Arg131 and hydrogen bonds with the side chains of Thr68 and Asp101 and the main chain carbonyl of Val55 (Figure 2C). Additional interactions include van der Waals and hydrophobic contacts as well as polar interactions mediated by water molecules.

Electron density is observed indicating the presence of a chemical moiety in the H-site that is attached to the thiol group of GSH that is bound to both monomers. The electron density feature is more prominent for the binding site of monomer A and has a shape consistent with a portion of the CBL moiety, conjugated with bound GSH (Figure 2B and Figure S1). For both monomers, there is no electron density for the carboxylate moiety of CBL and for monomer B, the remaining CBL density becomes progressively poorer going from the thiol group to the aromatic ring of CBL. These may be consequences of the high mobility of the CBL moiety due to its extensive exposure to the solvent and lack of specific interactions. The results of the molecular dynamics study discussed as part of this work, are consistent with this possibility. Furthermore, the average value of the final B-factors for CBL is 60.58 and for GSH is 27.42. Similar observations and conclusions were reported for hGSTA1-1 in the cases of ethacrynic acid-GSH and of S-hexyl-GSH complexes [43], [44]. Another possibility may be partial occupancy of the H-site by CBL.

Comparison of the structure with those of previously reported hGSTA1-1/GSH complexes shows very few differences in the position of the GSH atoms with the exception of the thiol group of GSH which rotates in order to accommodate the bond with the CBL moiety. However, the thiol group maintains an interaction with the catalytically important residue Tyr9. Several van der Waals and hydrophobic contacts exist between the CBL moiety and several residues including Gly14, Leu107, Leu108, Val111, Met208 and Phe220 (Figure 2C). The chloroethyl group of CBL appears to be relatively flexible and does not form any contact with protein residues. The CBL moiety is located in a position very similar to that of ethacrynic acid moiety in the hGSTA1-1/GSH-ethacrynic acid conjugate, albeit shifted by about 1 Å with respect to each other (PDB entry 1GSE), [43]. Comparison of the two structures shows few differences in the position of the protein side chains, with the exception of Phe10 side chain which rotates in order to form van der Waals contacts with the aromatic ring of CBL and significant differences in the positions of C-helix residues, in particular that of Met208 (Figure 2C). Interactions between CBL and the helix residues may be responsible for the apparent stabilization of the C-terminal helix H9 for monomer A, which in the structure of GSTA1-1 in complex with S-hexyl-GSH (PDB entry 1YDK) is not part of the model and is presumably disordered [44]. In the 1YDK structure, the same helix for monomer B was ordered due to packing interactions.

### Molecular dynamics simulations

To investigate the effects of ligand binding on the conformation and the dynamics of the enzyme, molecular dynamics simulations (MDs) of the apo hGSTA1-1 dimer were compared with those of the holo complex with the GSH–CBL adduct and the complex with GSH alone. The root mean square positional fluctuations of each Cα atom during the course of the MDs (Figure 3) indicate that binding of the GSH–CBL adduct results to an overall stabilization of the hGSTA1-1 dimer, as exhibited by the lower atomic fluctuations of the holo hGSTA1-1 complex with respect to the apo enzyme. This effect is more pronounced for helices H2 (residues 37 to 48) and H9 (residues 210 to 220), whereas the major part of H4 and H5 (residues 85 to 144) are slightly less mobile in the holo dimer (Figure 3). Interestingly, the same stabilization effect is evident in the MDs of hGSTA1-1/GSH complex, which exhibit a very similar mobility profile with respect to the hGSTA1-1/GSH–CBL complex (Figure S2). This observation indicates that binding of CBL at the hGSTA1-1/GSH complex is only marginally affecting the dynamics of the dimer.

**Figure 4:**
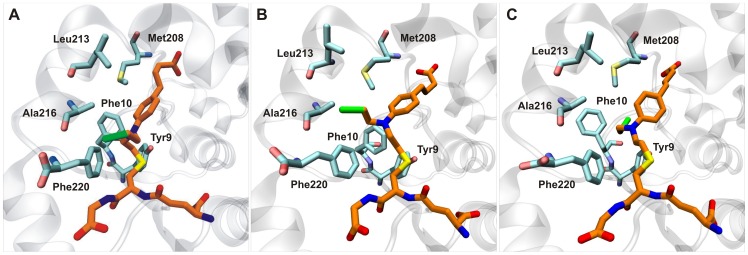
Snapshots from the 10-ns molecular dynamics simulation of the hGSTA1-1/GSH–CBL complex. (A) t = 0, (B) t = 2.1 ns and (C) t = 9.0 ns. Atom colors are cyan and orange for the C atoms of GST and GSH–CBL, respectively, blue for N, red for O, yellow for S and green for Cl.

As mentioned above, analysis of the MDs indicates that the CBL moiety is considerably more mobile than the GSH moiety, which is consistent with the progressively weaker electron density characterizing the CBL moiety (Figure 2B). In accordance, the root mean square deviation of the GSH atoms as a function of simulation time is evidently lower than that of CBL moiety (Figure S3). To illustrate the difference in the dynamics of the GSH–CBL adduct, three snapshots from the MDs of the holo hGSTA1-1 dimer are shown in Figure 4. The higher rigidity of the GSH moiety is evident throughout the 10 ns simulations in contrast to the CBL adduct, which exhibit major conformational changes. The chloroethyl moiety of CBL exhibits the higher mobility and is predicted to adopt several conformations that may even disrupt the π–π interactions of Phe10 and Phe220 (Figure 4C). The butyloxy moiety is predicted to be partially stabilized by a hydrogen bonding interaction with the backbone amide of Met208 (Figure 4B) and therefore exhibiting relatively lower fluctuations. On the other hand, the interacting residues from H9 are slightly perturbed by the high mobility of the CBL adduct and, in concert with H2, exhibit lower flexibility than in the apo enzyme.

### The interaction of CBL with hGSTA1-1 in the absence of GSH

#### Chemical modification of hGSTA1-1 with CBL

**Figure 5:**
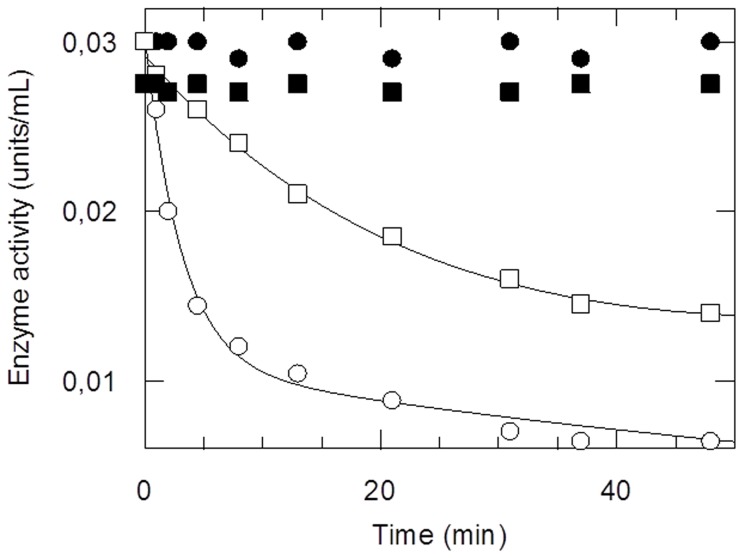
Time course of inactivation of recombinant hGSTA1-1 by CBL at pH 7. Enzyme incubated in the absence (•) or in the presence of 2 mM CBL (○). Enzyme incubated with 2 mM CBL in the presence of S-nitrobenzyl-GSH (1 mM) (□). Maleimide-modified enzyme incubated with 2 mM CBL (▪). At the times indicated, aliquots were withdrawn and assayed for enzymatic activity.

When hGSTA1-1 was incubated with CBL in the absence of GSH at pH 7, the enzyme was progressively inactivated (Figure 5), whereas in the absence of CBL, virtually no change in activity was observed when the enzyme was incubated under identical conditions. The inactivation of hGSTA1-1 by CBL was irreversible, and activity was not recovered either by extensive dialysis or gel filtration on Sephadex G-25. The kinetics of inactivation was biphasic (Figure 5), with the rapid reaction occurring immediately upon exposure of the enzyme to CBL followed by and the slow inactivation process to a final residual activity of approximately 22%. At all CBL concentrations used, biphasic kinetics were observed. The ability of specific ligands (e.g. substrates and inhibitors) to prevent enzyme inactivation by an irreversible inhibitor is usually taken as evidence that the inhibitor is either active site directed or affected by conformational changes resulting from ligand binding at the active site [30], [33], [46]. The effect of GSH analogue *S*-nitrobenzyl-GSH on the reaction of CBL with hGSTA1-1 was investigated (Figure 5). *S*-nitrobenzyl-GSH protects hGSTA1-1 from inactivation by CBL, indicating the specificity of the enzyme-CBL reaction.

**Figure 6:**
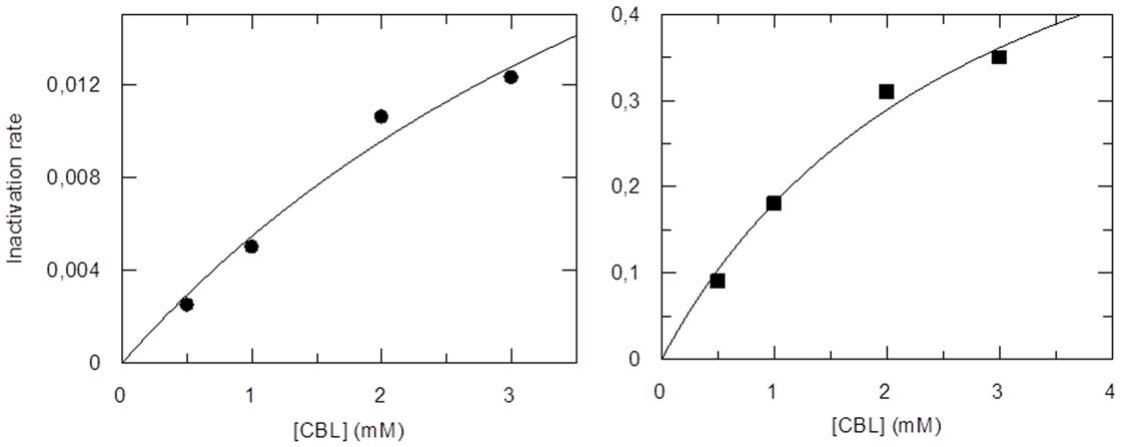
Dependence of the pseudo-first-order rate constant for the fast (▪) and slow (•) phase of inactivation on CBL concentration. hGSTA1-1 was incubated with various concentrations of CBL (0.5–3 mM) and the rate constants were calculated as described in the text.

The observed rate of inactivation for the fast and slow phases was dependent upon CBL concentration, as illustrated in Figure 6. This indicated that the reaction obeyed pseudo-first-order saturation kinetics and was consistent with reversible binding of reagent prior to covalent modification according to the following equation:
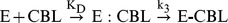
where E represents the free enzyme, E∶CBL is the reversible complex and E-CBL is the covalent product. The steady-state rate equation for the interaction is [30], [33], [34], [46], [47]:
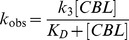
where k_obs_ is the observed rate of enzyme inactivation for a given concentration of CBL, k_3_ is the maximal rate of inactivation (min^−1^), and K_D_ is the apparent dissociation constant of the E∶CBL complex. From the data shown in Figure 6, K_D_ values of 2.95±0.5 mM and 6.05±0.8 mM, for the fast and slow reactions, respectively, were determined. Apparent maximal rate constants (k_3_) were determined equal 0.72±0.15 min^−1^ for the fast reaction and 0.04±0.01 min^−1^ for the slow reaction.

The stability of CBL against non enzymatic hydrolysis was demonstrated by measuring the rate of chloride ions release in conditions identical to those used in the enzyme inactivation experiments. The results showed that the first-order rate constant for CBL hydrolysis was 0.8×10^−5^ min^−1^. This corresponds to 0.0003% and 0.02% of the rate observed for the slow and fast phase, respectively. This suggests that the slow phase of inactivation observed is not due to the decomposition of CBL and is the direct result of CBL-enzyme interaction [46].

#### Stoichiometry and identification of hGSTA1-1 residue modified by CBL

**Figure 7:**
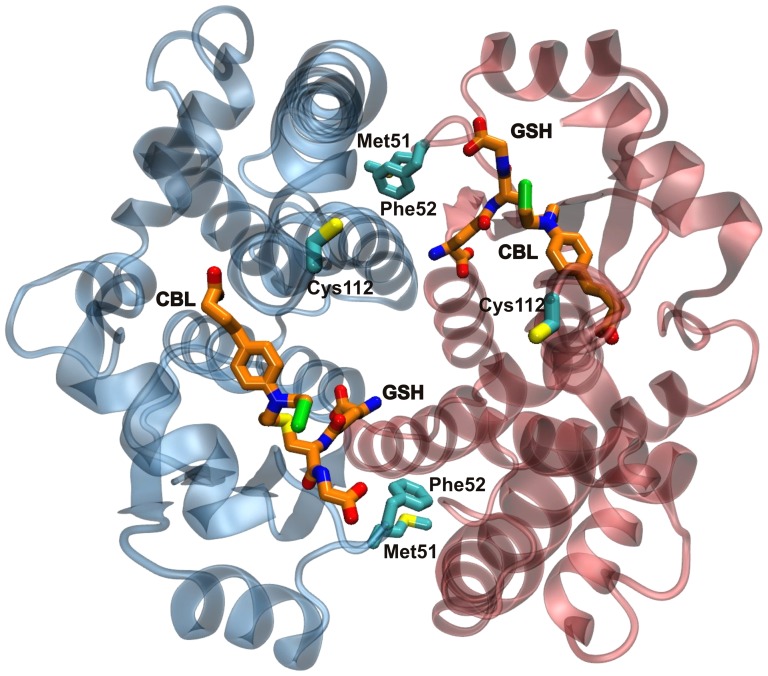
Structural representation illustrating the lock-and-key motif in hGSTA1-1 and a possible mode of communication between the two subunits. The key residues Met51, Phe52, Cys112 and the GSH–CBL adducts are shown in licorice representation.

To determine the stoichiometry of incorporation of CBL, modified and unmodified enzyme were subjected to MALDI-TOF MS analysis. The result of this experiment showed that the mass spectrum of unmodified enzyme gives a major peak centered at m/z = 25,482. The mass spectrum of CBL-modified enzyme shows an additional major peak whose mass is about 269 mass units higher (which is close to the mass of CBL) than that of the first major peak, which corresponds to the unmodified enzyme subunit. This indicates that only one of the two subunits is modified by CBL, while the other remains intact. In order to identify the amino acid residue modified, amino acid analysis and molecular modelling were employed. Direct amino acid sequence determination of the CBL-modified peptide was not possible due to its instability during Edman degradation reactions. Earlier findings [36] point out that Cys112 of hGSTA1-1 appears to be the most reactive residue against cysteine-reactive reagents, such as CBL. To analyse whether Cys112 is the nucleophile target for CBL, total cysteine determination using DTNB was carried out for the modified and free enzyme. The results from a total cysteine determination indicated that the modified enzyme shows loss of 0.87±0.21 mol of Cys/mol enzyme dimer, which is close to unity. The enzyme has one Cys residue per subunit (Cys112). From the analysis of the enzyme crystal structure it is evident that Cys112 lies adjacent to the hydrophobic substrate binding site and is located on the loop connecting helices H4 and H5 (Figure 7). Its side chain thiol is accessible for covalent modification by CBL and projects into the large, solvent-filled cleft which is widely reported in the literature to be the binding site of nonsubstrate ligands [45]. To provide further experimental evidence and establish the involvement or not of Cys121 in the reaction with CBL, chemical modification with maleimide was carried out on the basis of the known [36] susceptibility of Cys112 for modification by maleimides. The maleimide-modified enzyme retains most of its activity (92%), indicating that modification at Cys112 has little effect on the catalytic turnover of CDNB. The results of the present study showed that the maleimide-modified enzyme is resistant to inactivation by CBL, compared to the wild-type enzyme (Figure 5). Inspection of the electron density map in the vicinity of Cys112 for monomer B suggests the presence of multiple conformations for the H4–H5 loop. Although there is a density feature consistent with the shape and size of a CBL attached to Cys112, nevertheless the density is significantly fragmented and model building is not possible for that region of the structure.

## Discussion

Resistance to anticancer chemotherapeutic drugs remains a major obstacle in cancer chemotherapy [7], [8]. GSH is a critical determinant in tumor cell resistance to alkylating cytostatic agents. This has been attributed to the ability of GSH to compete with DNA for drug binding. The actual structures of these drug-GSH conjugates have been characterized for a number of these agents, such as melphalan, chlorambucil, and cisplatin [7], [38], [48], [49]. In the present work we showed that hGSTA1-1 interacts with CBL in two different ways, depending on the presence or absence of GSH. In the presence of GSH, CBL acts as an efficient substrate for hGSTA1-1. Kinetic analysis for GSTs has been reported for a vast number of compounds and the kinetic mechanism has been clarified [21], [50]–[52]. In general, kinetic mechanism of the GST-catalyzed conjugation reaction is very complex and class dependent. For example, several catalytic mechanisms, including random, ping-pong, and sequential, have been proposed [21], [50]–[52], but random binding order of substrates seems to prevail. In the case of hGSTA1-1 it has been demonstrated that the enzyme displays an all-of-the-sites reactivity for nucleophilic aromatic substitution reactions, whereas a half-of-the-sites reactivity has been suggested for addition reactions [53].

**Figure 8:**
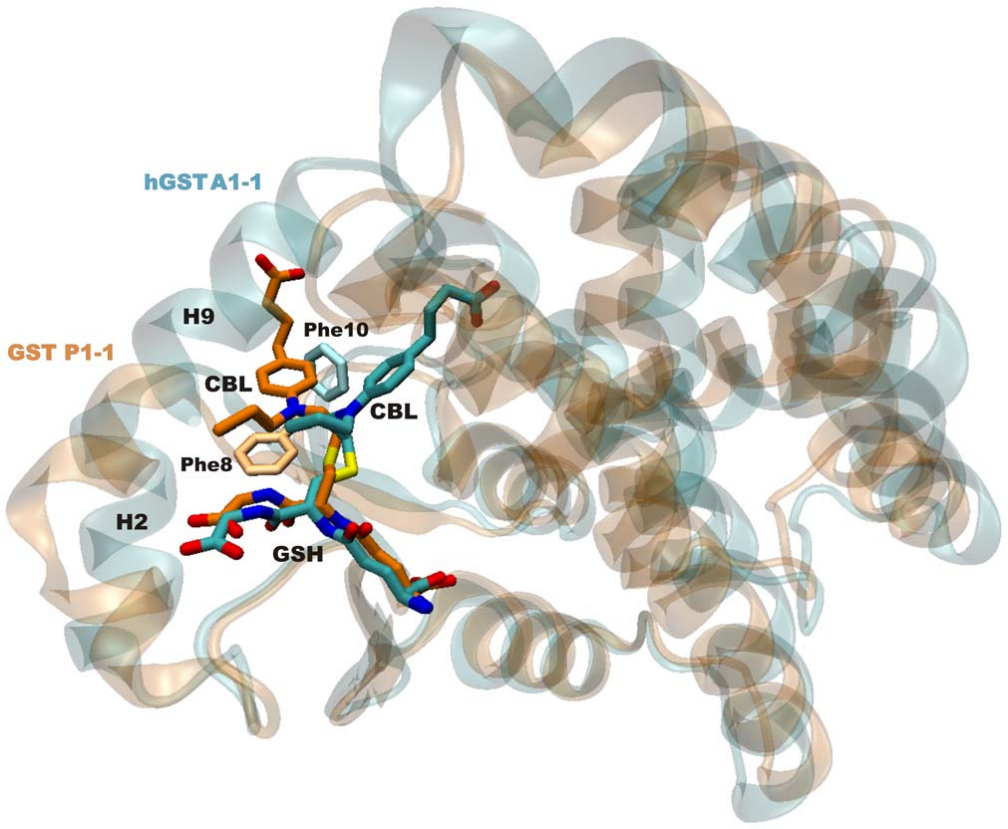
Superimposed crystallographic structures of hGSTA1-1 (PDB ID: 4HJ2, polypeptide ribbon and carbon atoms in cyan) and hGSTP1-1 (PDB ID: 3CSH, polypeptide ribbon and carbon atoms in orange) complexes with the GSH–CBL adduct. The interacting Phe10/Phe8 residue of hGST A1-1/P1-1 is shown for comparison.

The structure of another GST family member (human GSTP1-1) has been solved in complex with the GSH-CBL adduct (PDB entry 3CSH) [54]. In that structure, the CBL molecule is also partially exposed to the solvent, nevertheless it makes a few specific interactions with protein residues. Similarly to our structure, the Phe8 residue (corresponding to Phe10 of hGSTA1-1) is shifted towards the CBL molecule, thus enhancing π-π type interactions. However, when the two structures are superimposed it is evident that the CBL ring plane is located in different positions (Figure 8). This observation may be the consequence of significantly different H-site structures between the two isoenzymes due to the absence of conserved residues, and of a significant rotation of the small subdomain containing the H2 and H9 helices with respect to the large protein subdomain. In addition, it is well established that the function of the H-site is to accommodate xenobiotics at close proximity with the GSH cosubstrate, thus facilitating their enzyme-catalyzed conjugation. Due to its promiscuity for many different types of hydrophobic molecules, relatively shallow pocket shape and rare occurrence of polar residues, the H-site naturally does not feature a conserved binding mode for similar ligands. It is, therefore, expected that different GST classes are not likely to have preserved binding modes. In the GSH-CBL adduct, its high mobility and the absence of specific interactions between CBL and the enzyme suggest that the covalent bond formed between GSH and CBL is likely to be the main restraining factor for CBL in this position.

In the absence of GSH, CBL behaves as an irreversible inhibitor of hGSTA1-1. The enzyme inactivation by CBL exhibited biphasic kinetics although only approximately 1 mol of CBL per mol of enzyme was incorporated and only one catalytic site was modified in the enzyme dimer. In addition, compound *S*-nitrobenzyl-GSH that provided protection against inactivation, had the same effect for both phases of the inactivation. These observations imply that CBL binds at the same type of site during the reaction course. The biphasic kinetics observed may be explained by assuming that the two subunits, or at least the conformation or the dynamics of the Cys121 side chains of the subunits, are not equivalent towards the CBL reaction thus exhibiting different reactivity. In this context, Figure 3 depicts a plot of the RMSF (root mean square fluctuations) along the polypeptide chain, thus providing an indication of the relative flexibility of the different portions of the protein. Cys112 is located in one of the two regions with the highest mobility. The crystal structure shows that, while that region has well defined structure in monomer A, it is disordered in monomer B. While it is possible that differences in crystal contacts could account for such differences, one cannot rule out the possibility of an influence of the interaction between CBL and Cys112 on the monomer B. It is, therefore, reasonable to assume that conformational changes and changes in dynamics may account for the observed biphasic kinetics.

The active hGSTA1-1 enzyme is a homodimer. It has been a matter of debate whether the two monomers act independently or cooperatively in catalysis [36], [54]–[56]. An average incorporation of 1 mol of CBL per mol of enzyme dimer indicates that reaction of CBL with one Cys112 prevents the reaction of the Cys112 of the second subunit, a finding indicative of intersubunit communication. Several structures of homodimeric GSTs with bound products or product analogues have been published, showing no detectable asymmetry between the subunits. However, Grahn et al. [57] have shown that the C-terminal region can adopt an ordered helix-like structure even in the apo state, showing a strong tendency to unwind. In one subunit (subunit B) the C-terminal region in the apo structure is more ordered than the other, suggesting that the subunits are not equivalent. Subunit B seems better suited to bind a ligand, possibly activating the other subunit by conformational changes.

Steady-state kinetics with GSTs from several classes using CDNB and 1,2-dichloro-4-nitrobenzene as electrophilic substrates are consistent with the pattern of two noncooperative binding sites. However, active site titration of hGSTA1-1 with 1,3,5-trinitrobenzene (TNB) showed that maximal saturation of hGSTA1-1 occurs at 1 mol of TNB per mol of enzyme dimer [58]. Half-of-the-sites reactivity was also postulated for human GSTT2–2, based on stopped-flow analysis of pre-steady-state kinetic data [59]. Furthermore, the published crystal structure of the mouse mGSTA4-4 [60] and soy *Gm*GSTU4-4 [18] showed that, although GSH was bound at the active sites of both monomers, only one subunit appeared to be catalytically active at a time.

Analysis of the crystal structure of hGSTA1-1 provides a structural basis for the intrasubunit communication operating upon reaction of Cys112 with CBL (Figure 7). Although the H-sites of neighbouring subunits are distant, a plausible mode of communication between them is conceivable. Structural examination (Figure 7) reveals that the key residue bridging the dimer interface, Phe52, may play an important role in intrasubunit communication. This residue, together with Met51, forms the lock-and-key motif which is responsible for a highly conserved hydrophobic interaction in the subunit interface. These residues make contact with a hydrophobic patch on the alternate subunit, comprising in part of Met94, Phe136, and Val139. Since the interface contacts on the alternate subunit are largely found in a single kinked α-helix H_3_, the signal may be transmitted via the helix H_3_ to Cys112, which is located at the end of this helix. Conformational changes of this residue may abolish reaction of CBL with Cys112 at the second subunit. Thus, the observed intrasubunit communication is probably directed via Phe52 of the monomer-monomer contact region, to α-helix H_3_ of the adjacent subunit which contains Cys112. Misquitta and Colman [56], using wild-type-mutant heterodimers of hGSTA1-1 have shown that mutation of an amino acid residue in one active site affects the activity in the other active site. Modelling studies, moreover, showed that key amino acid residues and water molecules connect the two active sites and this connectivity is responsible for the cross-talk between the active sites [56]. These results are in concert with a ‘co-operative self-preservation’ mechanism, as proposed by Ricci et al. [61] for the human P1-1 isoenzyme. According to this mechanism, a co-operativity is utilized by the enzyme to provide self-preservation against inhibitors or physical factors, which threaten its catalytic ability. This mechanism is based on a structural intersubunit communication, by which one subunit, as a consequence of a modification, triggers a defence arrangement in the other subunit to prevent modification [61].

In conclusion, in the present work we investigated the interaction of the chemotherapeutic drug CBL with hGSTA1-1. Analysis of the crystal structure of hGSTA1-1/CBL-GSH complex showed that the CBL moiety, bound in the H-site, is partially ordered, exposed to the solvent making only a few specific interactions with the enzyme. Molecular dynamics simulations based on the crystal structure indicated high mobility for the CBL moiety and stabilization of the C-terminal helix due to the presence of the adduct. In the absence of GSH, CBL is shown to be an alkylating irreversible inhibitor for hGSTA1-1, modifying specifically Cys112 only in one subunit. These results suggest the existence of a structural communication between subunits, confirming that the two enzyme active sites are presumably coordinated.

## Supporting Information

Figure S1 **Fo-Fc electron density map contoured at 2.7σ showing electron density corresponding to bound GSH–CBL, superimposed on refined crystal structure.**
(TIF)

Figure S2 **Root mean square fluctuation (RMSF) of GST A1-1 Cα atoms calculated for the GSH (black) and the GSH–CBL (red) complexes from 10-ns molecular dynamics.** The graph shows the mean RMSF value from the two monomers.
(TIF)

Figure S3 **Root mean square deviations (RMSD) of GSH (black curve) and CBL moiety (red curve) atomic positions as a function of MD simulation time.** Top graph is for monomer A and bottom graph is for monomer B.
(TIF)
