# Supplementary figures and images for: The Interaction of the Chemotherapeutic Drug Chlorambucil with Human Glutathione Transferase A1-1: Kinetic and Structural Analysis
Source: PLoS One. 2013 Feb 27;8(2):e56337. doi: 10.1371/journal.pone.0056337 (PMC3584069; doi:10.1371/journal.pone.0056337)

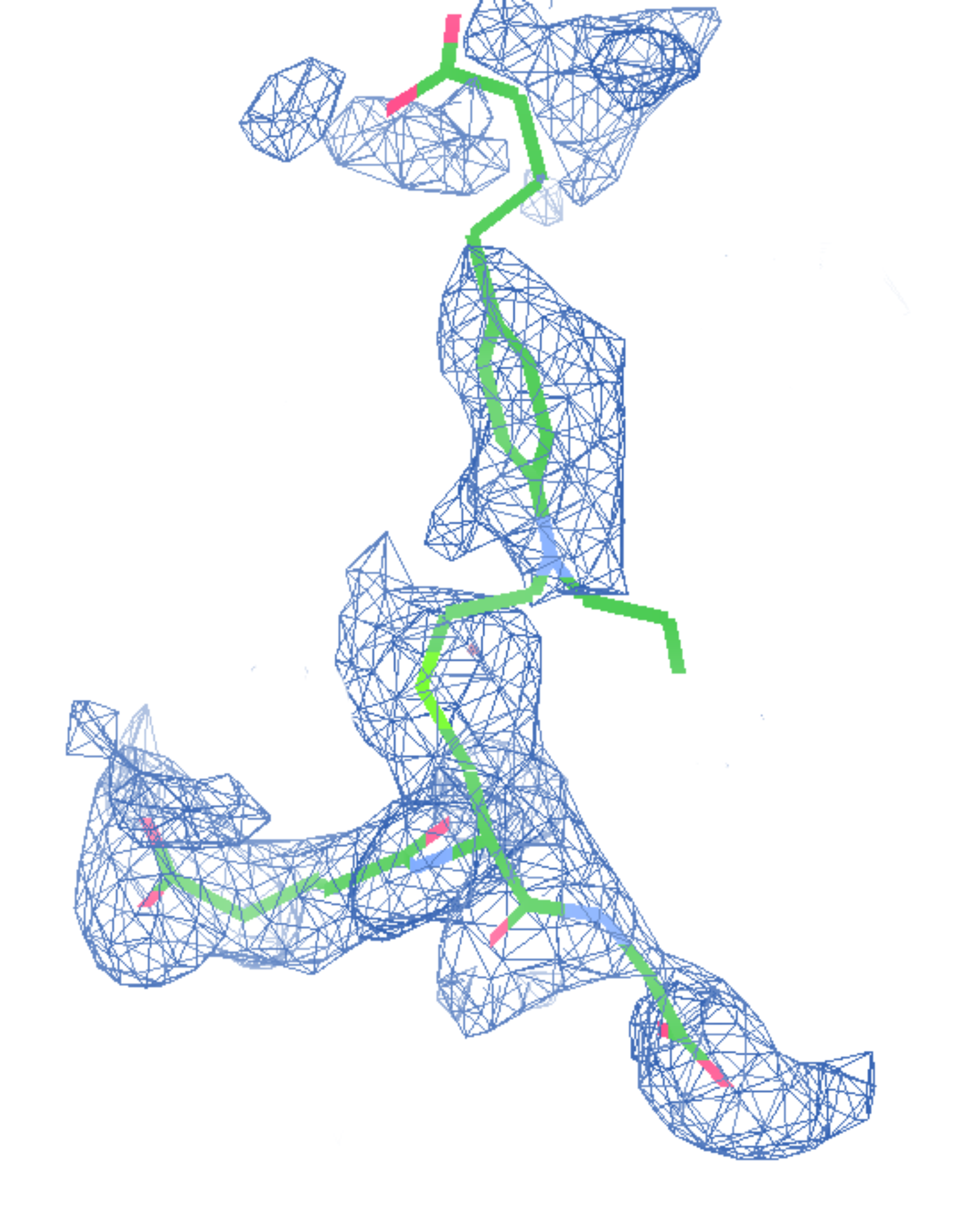

Supplement: Figure S1 — Fo-Fc electron density map contoured at 2.7σ showing electron density corresponding to bound GSH–CBL, superimposed on refined crystal structure. (TIF) [file pone.0056337.s001.tif]

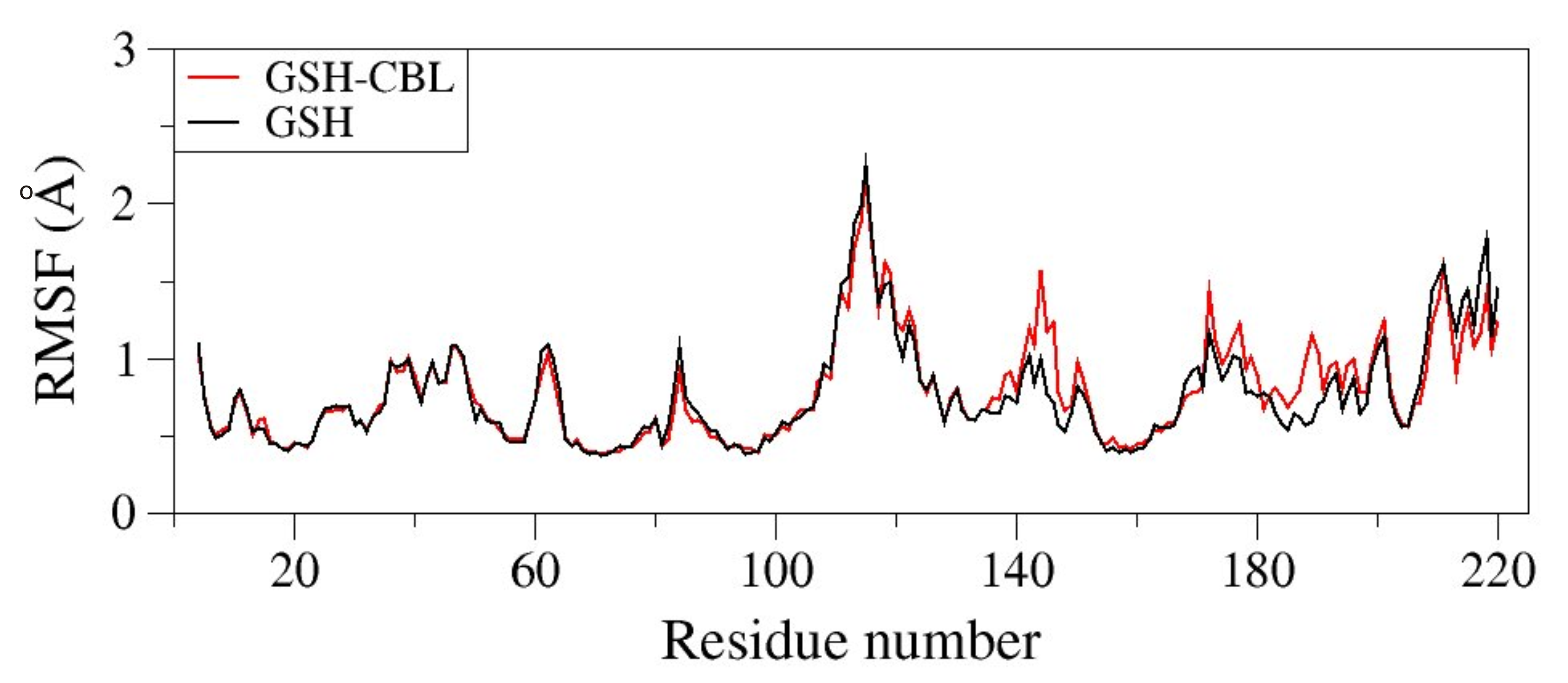

Supplement: Figure S2 — Root mean square fluctuation (RMSF) of GST A1-1 Cα atoms calculated for the GSH (black) and the GSH–CBL (red) complexes from 10-ns molecular dynamics. The graph shows the mean RMSF value from the two monomers. (TIF) [file pone.0056337.s002.tif]

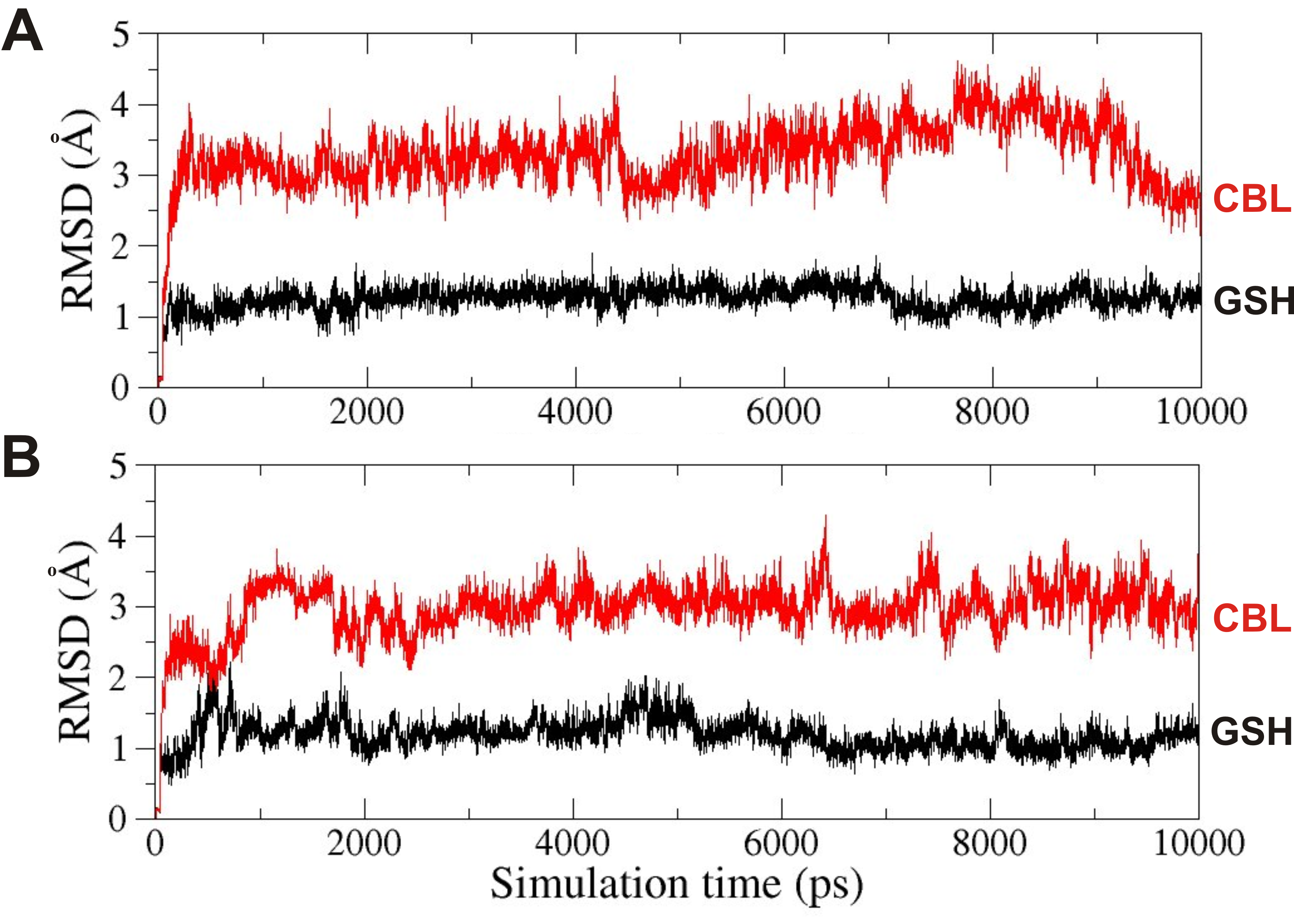

Supplement: Figure S3 — Root mean square deviations (RMSD) of GSH (black curve) and CBL moiety (red curve) atomic positions as a function of MD simulation time. Top graph is for monomer A and bottom graph is for monomer B. (TIF) [file pone.0056337.s003.tif]
